# Introduction and evolution of dengue virus type 2 in Pakistan: a phylogeographic analysis

**DOI:** 10.1186/s12985-015-0371-8

**Published:** 2015-09-22

**Authors:** Madiha Akram, Zareen Fatima, Mike A. Purdy, Amanda Sue, Sana Saleem, Irum Amin, Muhammad Shahid, Muhammad Idrees, Rabia Nawaz

**Affiliations:** Division of Molecular Virology, Centre of Excellence in Molecular Biology, University of the Punjab, Lahore, Pakistan; Centre for disease control and prevention, Atlanta, USA; Center for Applied Molecular Biology (CAMB), University of the Punjab, 87-West Canal Bank Road Thokar Niaz Baig Lahore-53700, Lahore, Pakistan

## Abstract

**Background:**

Pattern of Dengue periodic epidemics through the years along with sporadic cases of Dengue hemorrhagic fever followed by a severe 2011 epidemic of Dengue fever in Pakistan make Pakistan a Dengue endemic country. To study the entry and evolution of dengue virus serotype 2 (DENV-2) in Pakistan, we sequenced three full length genomes and 24 complete envelope sequences of DENV-2 from the years 2010, 2011 and 2013 collected from Punjab province of Pakistan.

**Methods:**

Phylogenetic and Bayesian phylogeographic analyses was applied to three full genome sequences as well as 24 envelope sequences to study the spatiotemporal dynamics of DENV-2 in Pakistan.

**Results:**

Most of the DENV-2 viruses from the years 2008 to 2013 formed a monophyletic Pakistani clade in IVb sublineage of cosmopolitan genotype except one 2008 DENV-2 strain. Phylogeographic analysis revealed that this 2008 DENV-2 strain was rooted to India 25.4 years ago with a location probability of 0.88. However Pakistani clade rooted back to Sri Lanka 12.6 years ago with a location probability of 0.57.

**Conclusion:**

DENV-2 genotype IV was introduced in Pakistan in two time events. First event was introduction from India to Pakistan in the late 1980s (around 1986), and second event was introduction from Sri Lanka to Pakistan around 2000. The later introduction event was responsible for major outbreaks in the Punjab region of Pakistan, including major 2011 outbreak. After the second Introduction event, DENV-2 circulated locally in the region forming a distinct Sublineage within the IVb cosmopolitan genotype of DENV-2.

**Electronic supplementary material:**

The online version of this article (doi:10.1186/s12985-015-0371-8) contains supplementary material, which is available to authorized users.

## Introduction

Dengue virus (DENV) having four serotypes (DENV-1 through 4) is a mosquito borne *Flavivirus* that has been found in more than 100 countries with estimated 50-100 million dengue infection cases each year [1]. The genome of DENV is a ssRNA with positive polarity and is approximately 11,000 nucleotides in length that encodes three structural proteins (capsid, membrane and envelope) and seven nonstructural (NS) proteins (NS1, NS2A, NS2B, NS3, NS4A, NS4B and NS5) [2]. In addition there are 5’ and 3’ noncoding regions (NCRs) of about 100 and 400 nucleotides respectively that form RNA secondary structures [3, 4].

Dengue fever in Pakistan was first reported in 1982 from Punjab since then at least eight small outbreaks of dengue fever had been reported from Pakistan [5–10]. A large epidemic of dengue fever hit Punjab in year 2011; in this outbreak the number of dengue infection cases was unusually very high. Dengue infection cases earlier reported were more than 15,000 and the count had increased to more than 50,000 patients in Lahore alone by the end of November 2011 [11]. Previous studies in Pakistan have reported circulation of all 4 serotypes of dengue virus, [10, 9] however, in this outbreak occurrence of serotype 2 was particularly higher with fewer cases of serotype 3 infections which were in concurrence with serotype 2. Punjab province was again under the threat of dengue during 2013, when 2165 dengue cases were witnessed by health departments in Punjab. This time most number of cases were from another main city, Rawalpindi [12]. Apart from it sporadic cases were also reported from other provinces like Punjab and Sindh. Dengue virus transmission in Pakistan has stretched across many of the major cities through all these year.

The serotype 2 of Dengue virus (DENV-2) has been a prominent serotype in many of these outbreaks especially 2011 outbreak. Phylogenetic analysis of partial DENV-2 sequences has revealed that genotype IV or cosmopolitan genotype of DENV-2 is circulating in Pakistan [10]. Phylogenetic analysis of complete genome sequences of Pakistani DENV-2 isolates has further added that it belonged to Indian subcontinent lineage of genotype IV or cosmopolitan genotype [13]. Another study pointed out that these Pakistani isolates were grouped together with Indian, Sri Lankan and Chinese isolates in sublineage IVb of cosmopolitan genotype [14]. A more detailed characterization of DENV- 2 isolated from Pakistan, however was needed to investigate spatiotemporal dynamics of DENV-2 in Pakistan.

Therefore to characterize the origin and emergence of DENV-2 in Pakistan we sequenced full length genomes of three DENV-2 samples and complete envelope regions of twenty four DENV-2 samples collected during 2010, 2011 and 2013 outbreaks in Pakistan and did Bayesian phylogeographic analysis on datasets of 59 full length and 137 envelope sequences of DENV-2 genome to characterize DENV-2 origin and transmission in Pakistan.

## Results

**Fig. 1:**
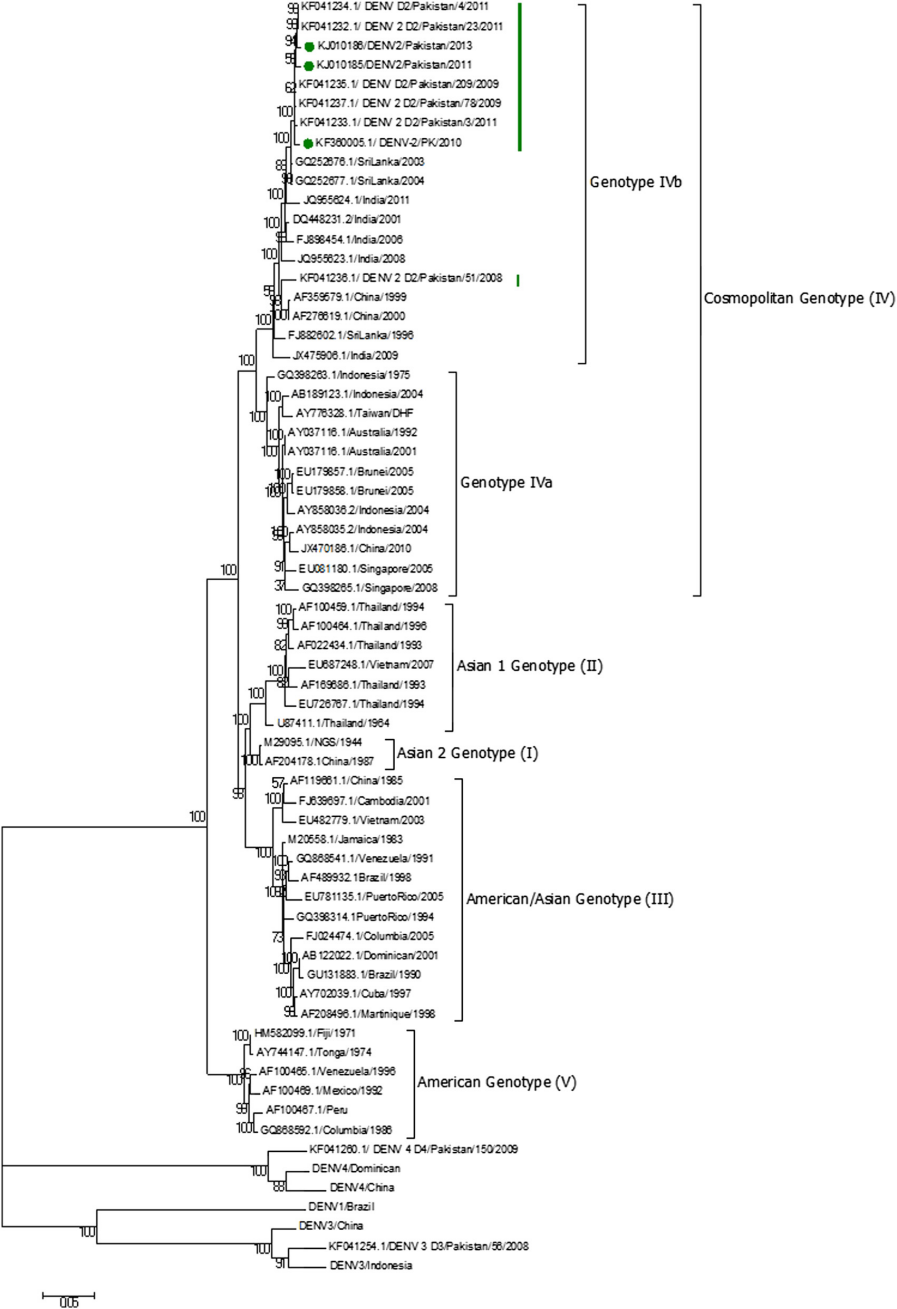
Phylogenetic Analysis of complete genome sequences of DENV-2 Pakistan. Evolutionary relationships of taxas represented in the form of phylogenetic tree computed by Maximum Likelihood Method using full-length genome sequences of 59 DENV-2 strains and DENV-1, 3 and 4 strains. The percentage of replicate trees in which the associated taxa clustered together in the bootstrap test (1000 replicates) are shown next to the branches. Pakistan strains sequenced in this study are marked with green dots. Genotypes I, II, III, IV and V of DENV-2 are also indicated. Sublineages of cosmopolitan genotype, IVa and IVb are indicated. Pakistani clade is highlighted with large green bar. And KF041236 strain is also marked with small green bar

**Fig. 2:**
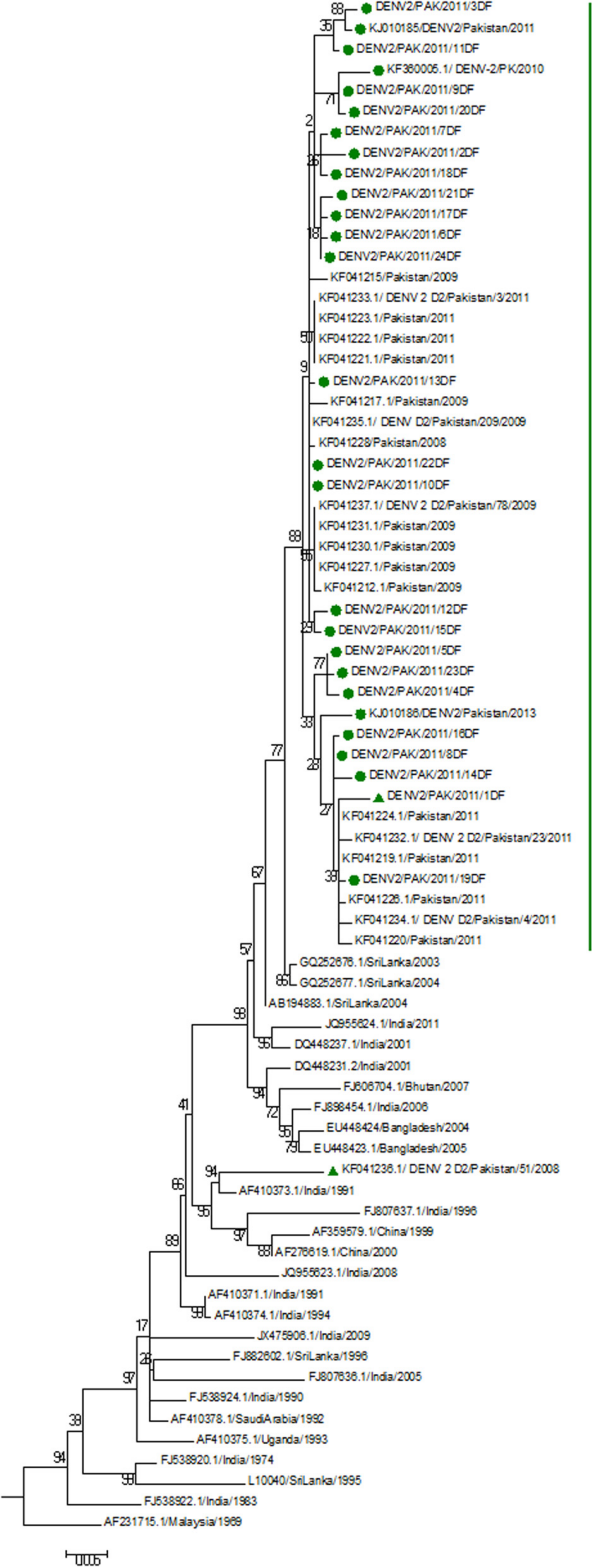
Phylogenetic analysis of envelope sequences of DENV-2 Pakistan. An enlarged view of cosmopolitan IVb clade. Evolutionary relationships of taxas represented in the form of phylogenetic tree computed by Maximum Likelihood Method using 137 envelope sequences of DENV-2 strains. Pakistani clade is indicated by a green bar. Isolates sequenced in this study are highlighted by green dots. KF041236 strain is highlighted by green triangle

Evolutionary divergence of DENV-2 as analyzed from phylogenetic trees generated from full length as well as envelope sequences grouped DENV-2 sequences in five genotypes (Fig. 1 and 2). Cosmopolitan Genotype IV of DENV-2 strains branched in to two distinct clades in which almost all of the Pakistani strains clustered together with South Asian countries including Indian strains collected after 2000 and 2003/2004 Sri Lankan strains. Sri Lankan DENV-2 isolates collected during 2003 and 2004 were ancestral to all the Pakistani isolates of DENV-2. However, one of the previously sequenced DENV-2 strain from Pakistan DENV-2/51/2008 grouped with Indian (1991 and 1996) and Chinese (1999 and 2000) isolates in another cluster (Fig. 2). The other clade of cosmopolitan genotype contained sequences from regions like Indonesia, Brunei, Taiwan, Singapore and Australia (Fig. 1).

### Phylogeographic analysis

**Fig. 3:**
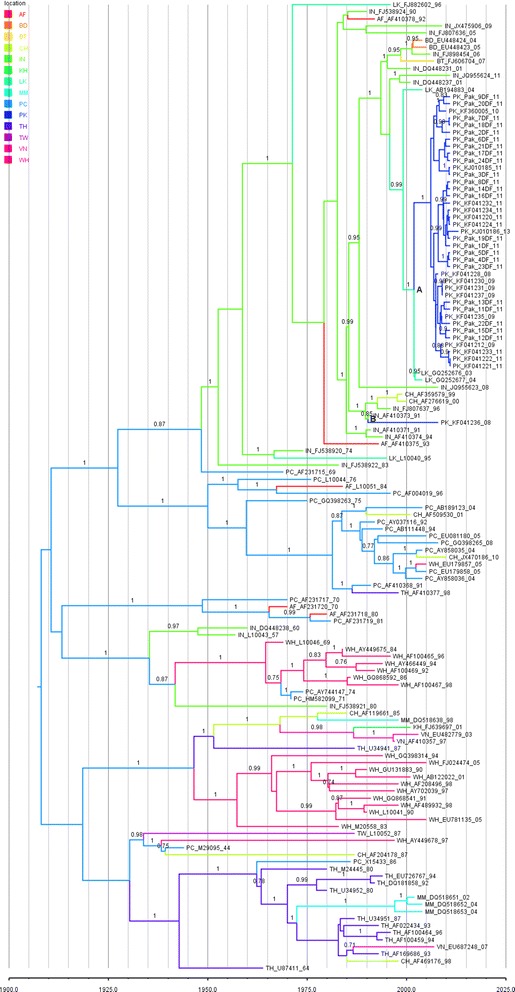
Discrete trait Bayesian tree. This tree was selected from the set of 12 discrete trait trees as having parameter estimates most similar to the mean estimates from all 12 trees. Colors represent locations. The location legend is in the upper left corner. The location codes are the same as in Additional file 5: Table S3. The numbers at the nodes are the posterior probabilities. The time scale is in calendar years. Sequences are identified as the two letter location code, accession number and the last two digits of the year in which the sample was collected. The letters A and B mark the locations of the MRCA node for the Pakistan clade and KF041236, respectively

To study the spatiotemporal dynamics of DENV-2 introduction in Pakistan, Bayesian phylogeographic analysis was applied on envelope (n = 137) sequences. An examination of the 12 consensus trees created by the discrete trait model showed that the Pakistani sequences form a monophyletic clade rooted by sequences from Sri Lanka. However, a single Pakistani sequence, KF041236, did not confine in Pakistani monophyletic clade and was grouped in a clade containing sequences from China and India (Fig. 3). The time to the most recent common ancestor (TMRCA) for KF041236 was 25.4 years ago (95 % HPD, 22.3 to 29.99 years ago). For the Pakistani clade the TMRCA was 12.6 years ago (95 % HPD, 10.3 to 15.5 years ago). The posterior probability for KF041236 and the Pakistani clade was 0.85 and 1.0, respectively.

KF041236 was rooted by sequences from India with a location probability of 0.88 for the Indian sequences as the root of the node. The Pakistani clade was rooted by sequences from Sri Lanka having a location probability of 0.57. An examination of the location probabilities for this node shows that Sri Lanka has the highest location probability (0.57, s.d. 0.06). The next highest location probability was 0.16 (s.d. 0.05) from Bangladesh (Additional file 1: Table S4).

**Fig. 4:**
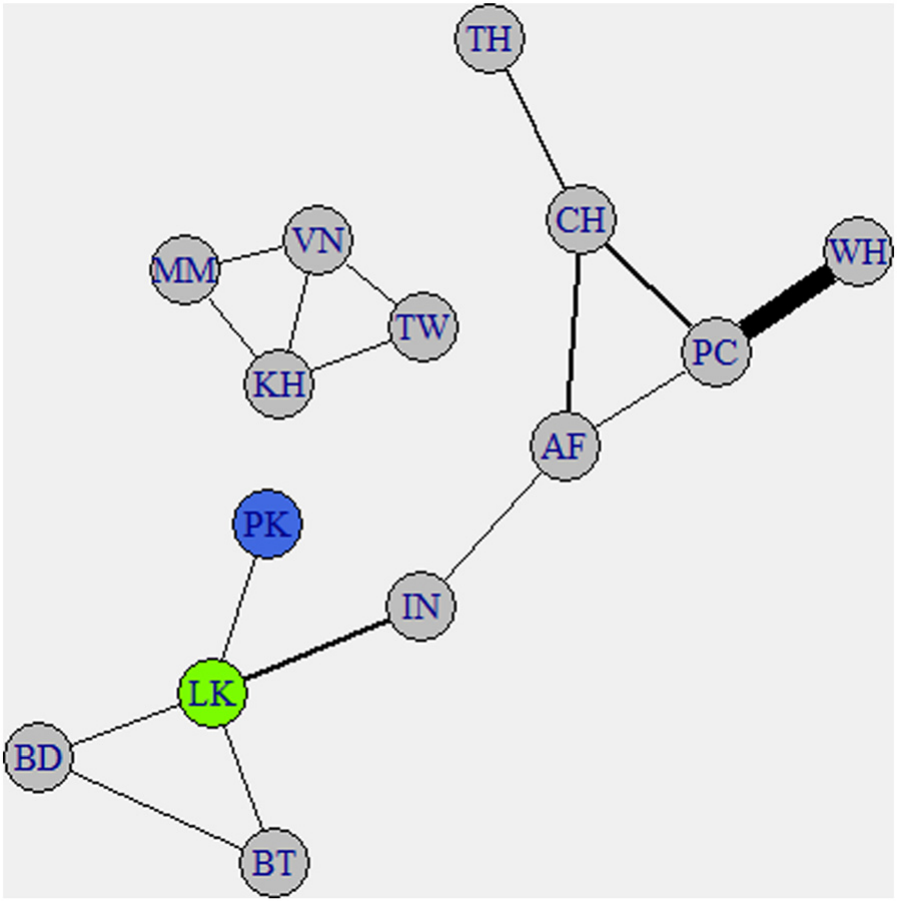
Network of linkages between pairs of locations as determined by SPREAD. The Pakistan node is colored blue, the Sri Lanka node is colored green, and all other nodes are grey. The thickness of an edge is related to the mean Bayes Factor for the location nodes connected to that edge. Linkages were only shown if that linkage appeared in at least 75 % of the 12 SPREAD analyses (Additional file 2: Table S5)

Spatial diffusion from the discrete trait rate matrix files was visualized using SPREAD to obtain linkages between pairs of locations as location indicators where the estimated Bayes Factor was greater than 3.0 (Additional file 2: Table S5). Like the phylogeographic trees the only significant linkage to the Pakistani sequences was to Sri Lanka (the blue and green nodes) (Fig. 4). The network contains two unconnected graphs. This doesn’t indicate that there is no connection between the two sets of locations, rather no significant linkage was found with a Bayes Factor of 3.0 or greater. Indeed linkages with Bayes Factors less than 3.0 will connect all the locations in the network. The linkage from KF041236 to India is not seen because of the bias due to the large number of Pakistani sequences in the Pakistani clade linked to Sri Lanka.

### Selection pressure analysis

No recombination event was detected by GARD. Significant selection pressure sites were those with a p-value < 0.1. A number of negatively selected sites were identified throughout the genome, however only six sites were found to be under positive selection (Table 1). There was one positively selected site in Pre-membrane (70), one in NS3 (10), one in NS4B (130) and three in NS5 (18, 440, 610).

**Table 1:** Sites of amino acid substitutions under positive selection computed by Mixed Effect model of evolution (MEME) analysis

Gene	Gene position	α	β^−^	q^−^	β^+^	P- value	Log L	KF041236	2010	2011	2013
Prm											
	70^£^	0	0	1.0	3916.54	.05	−8.79	S	S	A	A
NS3											
	10^¥^	0	<.0001	1	278.84	.00	−11.26	P	P	P	Y
NS4B											
	130^¥^	0	0	1	864.59	.04	−10.29	T	T	S	T
NS5											
	18^¥^	0	0	.14	1888.34	.00	−11.51	N	N	S	N
	440^¥^	<.0001	<0.0001	1.0	233.06	.09	−8.46				
	610^¥^	0	0	1	38.47	.08	−8.05	N	N	G	N

^¥^Amino acid substitution sites newly reported in this study.

^£^Amino acid substitution already reported in Pakistan [14].

α The maximum likelihood estimate (MLE) of the synonymous substitution rate α.

β^**+**^ The MLE of the unconstrained β non-synonymous rate.

β^**−**^The maximum likelihood estimate (MLE) of the non-synonymous rate for the branch class with β ≤ α.

LRT -Likelihood ratio test statistic for β^+^ = α (null) versus β^+^ unrestricted (alternative).

p- The p-value for the LRT test, using the mixture distribution: 0.33 χ^2^_0_ + 0.30 χ^2^_1_ + 0.37 χ^2^_2_

q- The q-value for independent tests (upper bound on the false discovery rate), derived from the corresponding p-value using Simes’ procedure.

## Discussion

Dengue outbreak in Pakistan emerged first in Southern city Karachi of Pakistan in 1994 [6], where serotypes 1 and 2 were major causative agents. During the years 2007 to 2009 this virus emerged in Northern regions of Pakistan, and this time serotypes 2 and 3 remained dominant [10]. A major outbreak hit Lahore city of Pakistan with serotype 2 being the causative agent of this outbreak and serotype 3 was less frequent. Subtype or genotype of Dengue virus serotype 2 (DENV-2) circulating in Pakistan during 2007-2009 was cosmopolitan as confirmed by phylogenetic analysis of C-Prm gene junction sequences [10]. In 2011 outbreak cosmopolitan or IV subtype of DENV-2 was confirmed with envelope and complete genome sequences. These sequences further grouped Pakistani sequences with Indian subcontinent lineage IVb [14]. Within this Indian subcontinent lineage IVb, Pakistani isolates formed a monophyletic clade [15]. To explore more evidences supporting DENV-2 dynamics in Pakistan that could possibly unravel the reason of major 2011 outbreak in this region, a detailed analysis on DENV-2 sequences was conducted. We performed a phylogenetic analysis and Bayesian phylogeographic analysis on a bigger data set of full length and envelope sequences including all the sequences from Pakistan from years 2008 till 2013.

Evolutionary evidences from phylogenetic analysis confirmed that Pakistani DENV-2 sequences grouped together in a monophyletic clade of Indian subcontinent lineage IVb of cosmopolitan genotype IV as suggested by other studies from Pakistan [14]. However one of the DENV-2 strain DENV-2/51/2008 (KF041236) clustered out of this monophyletic clade with Indian isolates of years 1991 and 1996. This suggested a different origin of this single isolate when compared to other isolates (Fig. 1 and 2). We therefore hypothesized that DENV-2 genotype IV might have emerged in Pakistan from two distinct origins.

Phylogeographic tree of envelope gene sequences also indicated that most of the Pakistani sequences formed a monophyletic clade, whereas, a single Pakistani sequence, KF041236, grouped together with Indian and Chinese isolates in a different clade (Fig. 3). It appears that the time to the most common recent ancestor (TMCRA) for KF041236 was about 25.4 years ago. This ancestor came from India which is already reported to be the ancestor of Cosmopolitan genotype [16]. It has been previously reported that Indian isolates collected from 1983 to 1991 shared a clade with isolates from Sri Lanka 1990, Uganda 1993 and China 1999 strains. This indicates that Indian isolates of cosmopolitan genotype (1983-1991) served as ancestors for the dispersal and distribution of this genotype to Sri Lanka, China and throughout the Indian Subcontinent [17]. Our results confirmed that these Indian isolates also travelled to Pakistan 25.4 years ago in 1980s, since this strain (KF041236) clustered with Indian isolates from the years 1991 and 1996 and Chinese strain from the years 1999 and 2000.

The other Pakistani clade has a more recent introduction into Pakistan. Its TMCRA was about 12.6 years ago and its ancestor probably came from Sri Lanka. This is supported by the location probability of 0.57 and 0.61 for Sri Lanka at the root of the Pakistani clade. Although this is a relatively low location probability it is 3.5 times greater than the next highest location probability (Bangladesh, Additional file 1: Table S4). Additionally, SPREAD analysis confirmed a significant linkage between Sri Lanka and the Pakistani clade in 92 % of the 12 discrete trait analyses run for the Dengue virus sequences (Fig. 4).

This second introduction times back to 2000. However, first DENV-2 outbreak in Pakistan came in 1994 in Karachi, this was followed by many outbreaks in the country suggesting that there must be an earlier event when DENV-2 was introduced in this region. The degree of severity of 2011 dengue outbreak also suggested an introduction of a novel strain of DENV-2 that has greater epidemic potential and fitness. Our analysis therefore suggested that the DENV-2 virus circulating in Pakistan was introduced in Pakistan from either India or Sri Lanka in two distinct time events. The first event appears to be introduction of the virus (KF041236 ancestor) from India to Pakistan around 1986 and the second appears to be introduction from Sri Lanka around 2000. We can infer that the virus that was introduced in a second event was better fitted in our region and therefore replaced the older DENV-2 virus since most of the Pakistani sequences from years 2011, 2009, 2010 and 2013 clustered in a distinct Pakistani clade.

It has been suggested that a lineage or virus subtype change might result from small changes in genetic makeup of virus and result in a virus with more fitness and virulence which can cause a bigger epidemic [18, 19]. We therefore analyzed all the full genome sequences from Pakistan for the amino acid substitutions. We detected a number of amino acid substitutions that were introduced in the sequences in Pakistani clade when compared with 2008 (KF041236) sequence, however Selection pressure analysis computed by Mixed Model of Evolution revealed that six amino acid sites were under positive selection pressure with a p value < .1 in complete genome of 9 Pakistani isolates from 2008 to 2013. One amino acid substitution in Pre-membrane region S70A, has already been reported as an amino acid substitution unique to DENV-2 2011 isolates in other recent studies on DENV-2 Pakistani isolates [14, 15]. In our study this site is under positive selection pressure with a p-value 0.05 and is consistent in most recent PK/2013 isolate. Apart from this, another polar (asparagine) to hydrophobic (glycine) amino acid substitution (N610G), in NS5 region of DENV-2, unique to PK/2011 strain sequenced in this study is significant with a p-value 0.08. This substitution is confined in the C-terminal domain of NS5, which is important for its RNA dependent RNA polymerase activity of NS5 protein in Flaviviruses [20] (Table 1).

Based on the genetic diversity between 2008 (KF041236) and rest of the sequences, difference in their time of introduction in the region, their origin and evolutionary dynamics, we can infer that introduction of DENV-2 in Pakistan 12 to 13 years back from Sri Lanka, replaced KF041236 DENV-2 strain and caused major outbreaks in the country including 2011 and 2013 outbreaks.

## Conclusion

We concluded that DENV-2 virus which remained a dominant serotype in all the major outbreaks has undergone two events of introductions in Pakistan; it first entered South of Pakistan from India in late 1980s and later entered in 2000 from Sri-Lanka. The product of later event then circulated locally forming a distinct Pakistani clade or sublineage, which was responsible for major 2011 outbreak.

## Methods

### DENV-2 Samples

27 DENV-2 isolates were included in this study (Table 2). All of them were collected from Punjab province of Pakistan during 2010, 2011 and 2013 outbreaks. RNA of dengue viruses was isolated from DENV-2 positive serum samples obtained from Molecular Epidemiology section of Molecular Virology Group CEMB that was collected during 2011 epidemic period and also during 2010 and 2013 outbreaks. The study was conducted in accordance with the 1964 Declaration of Helsinki and Guidelines for Good Clinical Research Practice in Pakistan. Institutional Review Board (IRB) of Post Graduate Medical Institute Lahore approved the studies.

**Table 2:** List of DENV-2 strains isolated in Pakistan, sequenced and analyzed in this study

No.	Strain	Clinical Manifestation	Year of isolation	City of isolation	GenBank Accession number
1	Pak_11DF_11	DF	2011	Lahore, Punjab	KP757116
2	Pak_12DF_11	DF	2011	Lahore, Punjab	KP757117
3	Pak_13DF_11	DF	2011	Lahore Punjab	KP757118
4	Pak_14DF_11	DF	2011	Lahore Punjab	KP757119
5	Pak_15DF_11	DF	2011	Lahore Punjab	KP757120
6	Pak_16DF_11	DF	2011	Lahore Punjab	KP757121
7	Pak_17DF_11	DF	2011	Lahore Punjab	KP757122
8	Pak_18DF_11	DF	2011	Lahore Punjab	KP757123
9	Pak_19DF_11	DF	2011	Lahore Punjab	KP757124
10	Pak_1DF_11	DF	2011	Lahore Punjab	KP757106
11	Pak_20DF_11	DF	2011	Lahore Punjab	KP757125
12	Pak_21DF_11	DF	2011	Lahore Punjab	KP757126
13	Pak_22DF_11	DF	2011	Lahore Punjab	KP757127
14	Pak_23DF_11	DF	2011	Lahore Punjab	KP757128
15	Pak_24DF_11	DF	2011	Lahore Punjab	KP757129
16	Pak_2DF_11	DF	2011	Lahore Punjab	KP757107
17	Pak_3DF_11	DF	2011	Lahore Punjab	KP757108
18	Pak_4DF_11	DF	2011	Lahore Punjab	KP757109
19	Pak_5DF_11	DF	2011	Lahore Punjab	KP757110
20	Pak_6DF_11	DF	2011	Lahore Punjab	KP757111
21	Pak_7DF_11	DF	2011	Lahore Punjab	KP757112
22	Pak_8DF_11	DF	2011	Lahore Punjab	KP757113
23	Pak_9DF_11	DF	2011	Lahore Punjab	KP757114
24	Pak_10DF_11	DF	2011	Lahore Punjab	KP757115
25	**PK/DENV-2/2010**	**DF**	**2010**	**Lahore Punjab**	**KF360005**
26	**PK/DENV-2/2011**	**DF**	**2011**	**Lahore Punjab**	**KJ010185**
27	**PK/DENV-2/2013**	**DF**	**2013**	**Rawalpindi Punjab**	**KJ010186**

Details of full length sequences are written in bold

### Complete genome and envelope gene amplification

Complete nucleotide sequencing of cDNA of full length DENV-2 genome of three patients derived DENV-2 isolates one from each year (2010, 2011 and 2013) and complete envelope gene (1485 bp) from 24 DENV-2 isolates was performed.

Nucleospin Viral RNA Extraction Kit (Macherey-Nagel, Germany) was used to extract viral RNA from 150 μl of serum sample according to the manufacturer’s instructions.List of primers used for the amplification and sequencing of overlapping fragments of DENV-2 genome are given in Additional file 3: Table S1. Primers were designed using Primer 3 online software based on complete sequences of DENV-2 listed in Additional file 4: Table S2. A cDNA copy of the viral genome was produced in a reverse transcriptase reaction using Superscript III reverse transcriptase enzyme (Invitrogen Biotechnologies USA) in the presence of anti-sense primers. Nested PCR was used to amplify complete DENV-2 genome in fragments. Taq DNA polymerase enzyme (Invitrogen Biotechnologies USA) was used to amplify smaller (≤1 Kb) DENV-2 genome fragments. Reaction mix was prepared as described by Fatima and colleagues [10]. The thermal profile for first round and second round using outer and inner primers respectively for each fragment was: initial denaturation at 94 °C for 2 minutes followed by 35 cycles of denaturation at 94 °C for 45 seconds, annealing at 52-55 °C for 45 seconds and extension at 72 °C for 2 minutes. A final extension was given at 72 °C for 10 minutes. Large amplicons of size 1.48 kb (ENV), 1.8 kb (NS3) and 2.7 kb (NS5), were amplified using Long PCR enzyme mix (Fermentas Life sciences USA). Reaction mix contained, 1 μl of 10 X long PCR buffer with 15 mM Mgcl_2_,1 μl of 2 mMdNTPs, 0.5 μl of 20 pm sense and antisense primers, 4.5 μl of dH_2_O, 2 μl of DNA template and 0.5 μl of 5U/ μl Long polymerase enzyme mix. The thermal cycler profile for both rounds was; initial denaturation at 94 °C for 2 minutes followed by 35 cycles of 94 °C for 20 seconds, 55 °C for 30 seconds, 68 °C for 2 minutes 30 seconds and final extension at 68 °C for 10 minutes.PCR products of different sizes were run on agarose gels of different concentrations depending upon the sizes of amplicons and gel purified using QIAquick Gel Extraction Kit (Qiagen, Germany) according to the manufacturer’s protocol. Eluted DNA was resuspended in 25 μl of distilled water and used as a template for sequencing.

### Sequencing reaction

Purified PCR products were then subjected to sequencing reactions according to Big Dye terminator cycle sequencing kit (Applied Biosystems, USA). The purified amplicons were sequenced in both directions using gene specific sense and antisense primers and analyzed on ABI PRISM 3100 Avant Genetic Analyzer (Applied Biosystems, Foster City, CA, USA). Sequencing PCR and ethanol precipitation was done by following Fatima and colleague’s protocol [10]. All the sequences were assembled using BioEdit sequence alignment editor (v 7.5.2) and submitted to GenBank with assigned accession numbers PK/2010-KF360005, PK/2011-KJ010185, PK/2013-KJ010186 for complete genome sequences and KP757106 to KP757129 for envelope sequences (Table 2).

In order to study the history and dynamics of Dengue virus evolution in Pakistan over the years we used both phylogenetic analysis and phylogeographic analysis.

### Phylogenetic analysis

We generated 3 full length and 24 envelope DENV-2 sequences in this study. We compiled two data sets of 59 DENV-2 complete genome sequences for complete genome phylogenetic analysis and 137 envelope sequences for phylogenetic and phylogeographic analysis (Additional file 4: Table S2). These DENV-2 sequences representing different parts of the world were downloaded from GenBank/EMBL/DDBJ online nucleotide sequence databases. Data sets also contained 6 full length and 14 envelope Pakistani sequences apart from the ones sequenced in this study, downloaded from online sequence databases. Sequences of DENV-1, 3 and 4 were used to root the tree. Envelope sequences used for Bayesian analysis from GenBank also had dates of collection (Additional file 4: Table S2). Alignment was done using Clustal W multiple alignment tool [21]. MEGA 5 programme was used for computing evolutionary analyses. Phylogenetic tree was constructed by using statistical Maximum Likelihood method [22]. Bootstrap support of 1000 was used for testing the robustness of trees.

### Phylogeographic analysis

To study the spatiotemporal dynamics of DENV-2 introduction in Pakistan, Bayesian phylogeographic analysis was applied on data set of envelope sequences of DENV-2. The sequence data set contained sequences from 34 countries. It was decided to group the sequences from some countries together to reduce the total number of locations in the model. Sequences from the Ivory Coast, Saudi Arabia, Senegal, Somalia and Uganda were grouped together as African sequences. Sequences from Australia, Brunei, Fiji, Indonesia, Malaysia, New Guinea, Singapore and Tonga were conflated as Pacific sequences. Sequences from Brazil, Columbia, Cuba, the Dominican Republic, Jamaica, Martinique, Mexico, Peru, Puerto Rico and Venezuela were grouped as Western Hemisphere sequences (Additional file 5: Table S3). This reduced the number of locations to 14.

### ModelTest

The best substitution model for the sequence alignment was determined in MEGA 6 [23] using the modeltest option, resulting in the TN93 substitution model with gamma rate categories and invariant sites as the best model.

### Clock rate

Beast (ver 1.8.2) was used to estimate the clock rate [24]. The strict clock and the uncorrelated exponential and lognormal clocks were used to estimate the clock rate using the TN93 + G + I substitution model with the coalescent constant size tree prior. Each clock model was run five times. The mean of these runs was determined and used as the clock rate for the specified clock. A stepping stone estimate for the log marginal likelihood [25] was obtained using the mean clock rates with the TN93 + G + I substitution model and the constant size tree prior. Each clock model was run as above except that the clock rate was fixed. The best clock was the lognormal clock (log marginal likelihood; -13205, -13223 and -13300 for lognormal, exponential and strict clocks, respectively). The mean lognormal clock rate was 7.01x10^−4^, which falls within the 95 % HPD for the clock rate estimated by Costa et al. [26].

### Discrete trait analysis

Beast was used to run the discrete trait model (http://beast-classic.googlecode.com/files/ARv2.0.1.pdf). The TN93 + G + I substitution model was used for the nucleotide sequences with the lognormal clock and a fixed clock rate of 7.01x10^−4^. The tree prior for this run was the GMRF (coalescent Gaussian Markov random field) Bayesian Skyride prior with the continuous time Markov chain rate reference prior distribution [27]. Location traits used the symmetric substitution model and BSSVS (Bayesian stochastic search variable selection) was used to infer the social network [28]. The default strict clock was used with an estimated clock rate and the default initial clock rate. 1x10^8^ iterations were used to reach an ESS value >200 in each run. The model was run 12 times. A 10 % burn-in was applied to each run. Parameter estimates were recorded as the mean and standard deviation for all 12 runs. Maximum clade credibility trees from TreeAnnotator (v1.8.0) were visualized using FigTree (ver. 1.4). The tree shown in Fig. 3 was selected from the set of 12 discrete trait trees as having parameter estimates most similar to the mean estimates from all 12 trees. Times to the most recent common ancestor are reported as “years ago” and all TMRCA estimates were calculated from the year 2013.

### SPREAD analysis

The discrete Bayes Factor for each pairwise location indicator between locations were calculated using SPREAD (ver. 1.0.6) with the default Bayes Factor cutoff of 3.0 [29]. Values greater than 3.0 were considered to be significant. Each run from the discrete trait models was analyzed in SPREAD. The network of linkages, where a link occurred between a pair of locations in at least 75 % of the SPREAD analyses, was created using igraph in R (http://cran.r-project.org/web/packages/igraph/igraph.pdf).

### Selection pressure analysis

Complete Genome sequences and envelope sequences were first screened for recombination events using GARD (genetic algorithm for recombination detection) tool [30]. Site specific selection pressure analysis was done on DENV-2 Pakistani isolates using Mixed Effect Model of Evolution (MEME) analysis [31]. Both the tools are available in HYPhy [32] an open source software package available under Data-Monkey Web server [33].

## Additional files

Additional file 1: Table S4. Location probabilities for the Pakistan clade. The top four location probabilities for each of the 12 discrete trait runs are listed by the two letter location code with the highest probability on the left. (XLSX 9 kb) Additional file 2: Table S5. Discrete Bayes Factors from SPREAD. Locations are denoted with two letter codes (Additional file 5: Table S3). The upper half of the matrix shows the mean (standard deviation) for the discrete Bayes Factor calculated for the 12 discrete trait models. The lower half of the matrix shows how many times a linkage had a Bayes Factor greater than 3.0. (XLSX 9 kb) Additional file 3: Table S1. List of primers used in amplification and sequencing. (XLSX 12 kb) Additional file 4: Table S2. List of complete sequences used in the analysis. List of sequences downloaded from GenBank/EMBL/DDBJ. Sequences are by their accession numbers, country of origin, location code and year of collection. Full length sequences used in phylogeography analysis are written in bold. (XLSX 15 kb) Additional file 5: Table S3. List of location codes used for phylogeographic analysis. A. List of two letter location codes. B. List of countries conflated into the WH, PC and AF location codes. (XLSX 8 kb)

## Abbreviations

DENV: Dengue virus
TMRCA: Time to the most recent common ancestor
HPD: Highest probability density
GARD: Genetic algorithm for recombination detection
MEME: Mixed effect model of evolution
PK: Pakistan
PCR: Polymerase chain reaction
cDNA: complementary DNA
MEGA: Molecular evolutuinary genetics analysis
GMRF: Coalescent Gaussian Markov random field
BSSVS: Bayesian stochastic search variable selection
